# Barriers to Timely Colonoscopy Completion Following Abnormal Fecal Immunochemical Test Results in a Safety-net System

**DOI:** 10.1007/s11606-025-10116-5

**Published:** 2026-01-20

**Authors:** Leslie Avilez, Jeanette Wong, Kristan Olazo, Shreya Patel, Rena Pasick, Ma Somsouk, Urmimala Sarkar

**Affiliations:** 1 Division of General Internal Medicine, Department of Medicine, University of California, San Francisco, CA USA; 2 Action Research Center for Health, Zuckerberg San Francisco General Hospital, University of California San Francisco, San Francisco, CA USA; 3 School of Medicine, University of California San Francisco, San Francisco, CA USA; 4 Division of Gastroenterology, University of California San Francisco, San Francisco, CA USA

**Keywords:** cancer screening, colon cancer, safety-net system

## INTRODUCTION

The effectiveness of stool-based colorectal cancer (CRC) screening is contingent on colonoscopy completion in patients with an abnormal fecal immunochemical test (FIT). Understanding the barriers that affect the follow-up of abnormal screening tests is essential for optimizing care in high-risk cohorts.^1^ Through an in-depth chart review, we describe the barriers contributing to incomplete colonoscopy follow-up within six months of an abnormal FIT in a safety-net system.

## METHODS

We conducted this retrospective chart review within an integrated safety-net system primarily serving low-income populations. The system includes 12 primary care clinics that refer patients to a single gastroenterology (GI) clinic. Using the electronic health record (EHR), we identified patients with an abnormal FIT between September 2021 and March 2023.

We manually reviewed 222 consecutive patient records using a standardized abstraction protocol. We located the FIT date and searched the EHR using the keywords “gastroenterology,” “colonoscopy,” “abnormal FIT,” and “positive FIT.” We continued abstraction until no new information was identified.

We developed a data extraction form in Microsoft Access to standardize data collection. The form included dropdown fields to categorize EHR-documented incomplete colonoscopy within six months of an abnormal FIT: (a) patient declined colonoscopy, (b) inability to reach the patient, (c) inadequate follow-up, and (d) comorbidities. When we identified other reasons, we documented them in a free-text box. All categories were mutually exclusive.

## RESULTS

We identified 728 patients with an abnormal FIT between September 2021 and March 2023. Of these, 65% (472/728) were referred to GI, 66% (311/472) of those who were referred were scheduled for a colonoscopy, and 84% (262/311) who were scheduled completed their colonoscopy within six months of the abnormal FIT date, or an overall six-month colonoscopy completion rate of 36%.

We conducted a detailed EHR review of 222 consecutive patients from among the 469 who did not undergo a colonoscopy. We found that 7% (*n* = 15/222) were scheduled for colonoscopy and 15% (*n* = 33/222) had completed their colonoscopy more than six months after their abnormal FIT. Of the 7% of patients with an abnormal FIT scheduled for a colonoscopy, 4% completed their procedure. Among those who completed their colonoscopy more than six months after their abnormal FIT, the median timeframe was 318 days (IQR: 254–451), or approximately 10 months.

The most frequently documented reasons for incomplete follow-up colonoscopy are listed in the Table 1: patient declined to complete colonoscopy (*n* = 44/222, 20%), inability to reach the patient (*n* = 42/222, 19%), and comorbidities (*n* = 17/222, 8%). Other EHR-documented reasons included that patients were no longer part of the health system, had completed FIT in error, or were deceased by the time of review. Some patients had no documented reasons for incomplete follow-up colonoscopy.

**Table 1:** EHR Documented Reasons for Incomplete Follow-up Colonoscopy Within 6 Months

Categories Identified	Total *N* = 222 cases, *N* (%)
Patient declined colonoscopy	44 (20%)
Inability to reach the patient	42 (19%)
No documented reasons	35 (16%)
Completed colonoscopy more than 6 months after FIT date	33 (15%)
Not in the health network	18 (8%)
Comorbidities	17 (8%)
Scheduled for colonoscopy	15 (7%)
Screened in error^a^	14 (6%)
Deceased	4 (2%)

^a^Screened in error refers to FIT tests performed for indications other than their intended purpose for colorectal cancer screening, as identified by chart review

## DISCUSSION

We conducted a retrospective chart review to characterize EHR-documented reasons for incomplete follow-up colonoscopy within six months of an abnormal FIT. Consistent with prior studies, colonoscopy completion within six months after an abnormal FIT was inadequate within this safety-net system. Another safety-net system study found that 42% of their study population did not undergo a follow-up colonoscopy within a year of an abnormal FIT.^2^ In our analysis, the most commonly EHR-documented reason for incomplete follow-up colonoscopy was that the patient declined the procedure, consistent with findings from other studies.^3,4^ This suggests a need for enhanced communication between healthcare providers and patients to improve adherence to CRC screening. Strategies include improving patient education about the benefits of screening and ensuring that patients are willing, understand the process, and can complete screening.^5^

This review was designed to inform ongoing efforts to understand and improve colonoscopy completion within the safety-net setting. Some patients with delayed follow-up may have been diagnosed with CRC outside our system or without a recorded colonoscopy, so CRC cases may be underestimated. Building on these findings, we developed a multilevel intervention to enhance colonoscopy completion, as described in our protocol.^6^ Given the required coordination among primary care, patient, and specialty care, we expect that an intervention encompassing direct patient communication and engagement, provider feedback, and improvement of clinic processes will be necessary to optimize colonoscopy completion.

Future research should investigate patients who declined colonoscopy and those without documented reasons for incomplete follow-up to better understand barriers and inform targeted interventions.

## Data Availability

The datasets generated during and/or analyzed during the current study are not publicly available due to the inclusion of sensitive personal data and associated ethical restrictions but are available from the corresponding author on reasonable request.
